# Nano-Photocatalytic Materials: Possibilities and Challenges

**DOI:** 10.3390/nano11030688

**Published:** 2021-03-09

**Authors:** José M. Doña-Rodríguez, Elisenda Pulido Melián

**Affiliations:** Grupo de Fotocatálisis y Espectroscopia para Aplicaciones Medioambientales (FEAM. Unidad Asociada al CSIC por el Instituto de Ciencias de Materiales de Sevilla), Departamento de Química, Instituto de Estudios Ambientales y Recursos Naturales (i-UNAT), Universidad de Las Palmas de Gran Canaria, 35017 Las Palmas, Spain; jose.dona@ulpgc.es

Photocatalysis is one of the most promising processes within catalysis, due to its increasing potential and the possibility of its being combined with renewable solar energy. There are countless applications, such as hydrogen production from wastewater, decontamination and disinfection of gaseous and water effluents, and more specific applications such as autocleaning surfaces, biosensors, or new chemical synthesis pathways. Photoactive semiconductor nanomaterials form the basis of all of these, catalyzing reactions by their capacity to photogenerate charge carriers.

Photocatalysis has progressed slowly and has established itself in the worldwide market in certain applications. Small-capacity reactors, paints and cements to decontaminate and disinfect are some examples. Despite this progress, the synthesis of even better materials is needed to increase activity and reduce costs and, thus, achieve a solid commercial development.

The aim of the Special Issue “Nano-Photocatalytic Materials: Possibilities and Challenges” was to collect the latest contributions that aim to overcome the drawbacks that photocatalysis still exhibits and that prevent its large-scale development.

Most works focused on the development of better materials, referring mainly to an increase in the time of photogenerated charge separation and to the widespread useful range of the spectrum for degradation. These materials were mainly tested for the degradation of dyes and emerging compounds, although some references worked with gases and others developed new synthesis pathways.

Enesca and L. Andronic [1] stated in a mini-review that improvements in the structures of the photocatalysts could lead to the optimization of energy consumption and the sustainability of the process. In addition, they claimed an urgent need for standarization of the photoactivity results in order to be able to carry out serious comparison studies that could place photocatalysis in large-scale implementation. Catalysts composed by heterostructures have caught the attention of the recent works in order to improve charge separation and light absorption. Four different mechanisms are possible in the heterostructures: p-n junctions, Schottyky junctions, type II heterostructures and Z-scheme heterostructures. H. Zhang et al. [2] studied the effect of different crystal phase contents in the photoactivity of TiO_2_ nanofibers obtained by electrospinning. Anatase-rutile junctions give place to a type II band alignment facilitating efficient electron–hole separation. A.A. Yaqoob et al. [3] reported a review about the sate-of-the-art of GO-based ZnO nanocomposites. This review focused on synthesis procedures and photoactivity mechanism from a critical point of view; the potential for applications considering stability, reusability and recyclability was also discussed. GO-based ZnO, type II heterostructure, exhibits favorable characteristics in both visible-light absorption and charge separation. Y.C. Liang and Y.C. Liu [4] reported the synthesis of core-shell structures based in TiO_2_ nanorods and a visible sensitizer, a narrow bandgap oxide. TiO_2_ nanorods were synthesized by hydrothermal method and covered by ZnFe_2_O_4_ by sputtering deposition. The junction of TiO_2_ and ZnFe_2_O_4_ leads to the formation of type II band structure. Sensitization has been considered an efficient approach for developing visible-light-responsive photocatalysts. On the other hand, J.J. Murcia et al. [5] opted for dye sensitization with quinizarin and Zinc protoporphyrin. The sensitizers were incorporated during the sol-gel synthesis of TiO_2_ powder, which, afterwards, was submitted to an alkaline treatment to obtain TiO_2_ nanotubes. They concluded that the sensitization is not always beneficial for the degradation of pollutants; for example, positive results were obtained for methyl orange but not for phenol. J. Yang et al. [6] also synthesized nanotubes that exhibited good activity for the degradation of methyl orange. However, the synthesis method was different: in this case, a Ti foil was subjected to alkaline hydrothermal treatment.

Another alternative to obtain visible-light-assisted reactions is through the combination of semiconductor and noble metal nanoparticles; these are the so-called plasmonic photocatalysts. Z. Wei et al. [7] discussed the influence of the morphotolgy of the TiO_2_-based photocatalyst to control electron transfer to noble metal nanoparticles (gold, silver or platinum). The efficiency under visible irradiation also is conditioned by the nature, oxidation state and photoabsorption properties (plasmon resonance) of the metal. Although in studies of contaminant degradation, there is not a single better noble metal for whatever the conditions, in the case of antibacterial applications, silver was exhibited to be the best one. A. Wafi et al. [8] obtained nitrogen-doped TiO_2_ catalysts with silver photodepostited on its surface that exhibited good activity results in both degradation of organic compounds and disinfection tests.

All of the previously mentioned works described simple synthesis methods. According to A. Belet et al. [9], sol-gel synthesis of TiO_2_-based materials is suitable to obtain the most promising photocatalysts for a scaled-up application due to their simplicity and cost. They studied both organic and aqueous synthesis pathways, with those materials obtained with organic solvents being more active. In addition, the photocatalysts were deposited as thin films on glass slides by dip-coating procedure. The support of photoactive materials brings significant savings in respect to the removal of the catalyst from the reaction media.

There is great concern about the degradation of pharmaceutically active compounds such as antibiotics: these are not degraded in conventional wastewater treatment plants and they are discharged into the environment. Photocatalysis has shown its power in this challenge, and several works of this Special Issue test their materials with this kind of probe molecule [1,9,10,11]. J. Patel et al. [10] synthesized ZnS quantun dots doped with manganese to degrade a fluoroquinolone antibiotic, norfloxacin, while H. Zhang et al. [11] combined a UiO-66-NH_2_ metal–organic framework (MOF) with g-C_3_N_4_ and CdS to eliminate tetracycline. MOFs have attracted increasing attention due to their many advantages, such as high specific surface area, tunable porous structure and large number of active sites.

Photocatalysis is a technique that, on many occasions, needs to be combined with other physical processes to improve the pollutant removal efficiency. In this sense, P. Pham et al. [12] synthesized materials with adsorptive and photochemical properties. These materials are based on magnetite (MNP) covered by humic acid substances (HA). The magnetite also serves as a non-active support that favors photocatalyst recovery from reaction media due to its magnetic characteristics. The article explores the photocatalytic mechanisms of HA-MNP to treat water contaminated with As(III).

The reactions promoted by photocatalysis can constitute new synthesis pathways. G. Zuo et al. [13] studied the synthesis of hydrogen peroxide under visible light with heterostructures that consisted of graphitic carbon nitride (CN) and a Au co-catalyst. They focused on the modification of this heterostructure with β-cyclodextrin to improve the hydrophobicity as well as oxygen adsorption of CN. Those improvements led to increasing efficiency because H_2_O_2_ was directly formed via a two-electron oxygen reduction reaction.

Finally, two works dealt with the degradation of gaseous contaminants, specifically those exhausted from automobiles that are important contributors to urban air pollution. Both studies were carried out with the reference commercial photocatalyts, Aeroxide TiO_2_ P25. M.J. Hernández Rodríguez et al. [14] focused on mechanism issues. They observed that the presence of palladium particles on the TiO_2_ surface favors NO removal. This is because palladium particles modify the interaction of water molecules with the catalyst surface. On the other hand, G. Luo et al. [15] carried out a study from a practical point of view. TiO_2_ was sprayed in pervious concrete that was tested with real automobile exhaust source and NOx and hidrocarbons were monitored.

The Special Issue gives an overview of the material strategies used in photocatalysis, its fundamental applications in the degradation of pollutants from aqueous and gaseous effluents and the development of new synthesis pathways. The guest editors are very satisfied with the contributions received and finally published.
